# Electropriming of wheatgrass seeds using pulsed electric fields enhances antioxidant metabolism and the bioprotective capacity of wheatgrass shoots

**DOI:** 10.1038/srep25306

**Published:** 2016-05-05

**Authors:** Sze Ying Leong, David John Burritt, Indrawati Oey

**Affiliations:** 1 Department of Food Science, University of Otago, Dunedin, New Zealand; 2 Department of Botany, University of Otago, Dunedin, New Zealand

## Abstract

The influence of pulsed electric field (PEF) (0.5–2 kV/cm) treatment of wheatgrass (*Triticum aestivum* L.) seeds, with different water contents, on antioxidant metabolism in the resultant seedlings was investigated. Imbibing seeds to a water content of 45% or greater prior to PEF treatment increased the glutathione level and activities of superoxide dismutase, catalase, glutathione reductase, glutathione peroxidase and ascorbate peroxidase in the resultant seedlings, compared to untreated controls. Pre-culture of human intestinal Caco-2 cells with simulated gastrointestinal digests of electrostimulated seedlings enhanced the ability of Caco-2 cells to cope with H_2_O_2_-induced oxidative damage, determined by the 3-(4,5-dimethylthiazol-2-yl)-2,5-diphenyltetrazolium bromide (MTT) and lactate dehydrogenase (LDH) release assays. The Caco-2 cell MTT and LDH assays correlated better with the increases in seedling glutathione content and antioxidant enzyme activities compared to the 2, 2-diphenyl-1-picrylhydrazyl (DPPH) total antioxidant capacity assay, an assay commonly used to determine the ability of plant extracts to protect cells from oxidative damage. These results demonstrate for the first time that PEF treatment of imbibed seeds can stimulate changes in metabolism in the resultant seedlings, increasing the bioprotective potential of their shoots/sprouts and hence value as functional foods.

In many developed countries there is an increasing awareness among their populations that a healthy diet is important for the prevention of disease, mitigation of the symptoms associated with ageing and an improved quality of life^1^,^2^. As a result there is an increasing demand for functional foods, particularly those of natural origin. While the partnering of traditional plant breeding with molecular marker-assisted breeding techniques and, when required, genetic engineering approaches are important for the production of high-quality nutritious plant-based foods^3^, or the raw material from which such foods can be produced, crop management, postharvest storage and processing systems are also important. These novel approaches would provide additional opportunities to manipulate the functional characteristics of plant-based foods^4^,^5^.

The sprouts of germinating seeds (primary shoots) of many crop plants have been used for food since before recorded history. In recent years sprouts have been found to contain high levels of bioactive metabolites, making them good candidates for functional foods^6^,^7^. Because of this the demand for high quality sprouts has increased. However, the production and marketing of sprouts as functional foods may be hampered by variations in the levels of bioactive metabolites, with levels very susceptible to batch variations. To overcome these problems several recent studies have investigated how growth conditions can be manipulated and chemical elicitors, such as chitosan and H_2_O_2_, can be used to increase the production of bioactive metabolites in the sprouts of existing cultivars^8^,^9^. Chemical elicitation has proven to be an effective method to enhance bioactive metabolite production, as with many commonly used agricultural chemicals, however supplying a uniform dose to each seed/seedling can be problematic. In addition, many consumers could perceive application of chemical elicitors to functional foods as being undesirable.

Treatment of plant-based food products with pulse electric fields (PEF) is a commercially used food processing technology that can be apply in a batch mode or in a continuous mode that allows large volumes of material to be reliably processed. It is an energy efficient, non-thermal, minimal processing technology that can be used to change the structural characteristics of plant-based foods, facilitate the release of bioactive metabolites and/or increase the shelf life of plant-based foods, without the use of chemicals^10^. PEF is considered a gentle, sub-lethal membrane permeabilisation technology, which relies on the exposure of the plant material to an external electrical field, in the form of short repetitive high voltage pulses, at a sufficient level induces the transient or permanent formation of pores in cell membranes^11^.

PEF treatment of seeds has been shown to influence the growth responses of *Arabidopsis thaliana* seedlings^12^, lettuce seedlings^13^, germinating barley grains^14^ and the seedlings of several other plant species^15^. Although the mechanism(s) by which seeds and seedlings respond to PEF is still largely unknown, the stimulatory or inhibitory growth effects induced by PEF are dependent on both the intensity of the electric field strength used and the physiological state of the seeds prior to treatment. Since the processes of seed imbibition and germination involve a series of major physiological and biochemical changes, PEF technology could potentially be used to manipulate these processes, which could result in seedlings with different nutritional properties and metabolite compositions, compared to seedlings from untreated seeds.

The excess production of reactive oxygen species (ROS) is a common response in plant cells under stress^16^,^17^. There is evidence demonstrating that the permeabilisation of plant cell membranes caused by PEF treatments promotes the excessive production of ROS and that this imbalance between ROS production and the capacity of endogenous antioxidants to neutralise the ROS lead to oxidative stress^18^. Plants under oxidative stress often have elevated levels of antioxidants and other protective metabolites, many of which promote health in humans^19^. However, to date, there are no definitive studies that address whether PEF-induced ROS production/accumulation does in fact influence antioxidant metabolism in plant cells in a positive or negative manner or if mild PEF treatments could increase health-promoting properties of some plant-based foods.

Wheatgrass (*Triticum aestivum* L.) is a widely used health food, consumed most often as fresh juice or as tablets, capsules and liquid concentrates. Wheatgrass formulations have been shown to possess various pharmacological properties such as antioxidant^20^, anti-cancer^21^, anti-diabetic^22^ and neuro-protective^23^ activities. In this study we investigated if exposure of imbibing wheatgrass seeds to non-lethal PEF treatments could trigger a stress response in the resultant seedlings, and therefore create a functional food with an enhanced bioprotective capacity. To achieve this we investigated the key factors that could potentially influence the response of wheatgrass seeds to PEF, e.g. the water content of the seed and the PEF electric field strength applied. To assess the impacts of PEF on the resultant seedlings we monitored seedling growth and the levels of key antioxidant markers, namely vitamin C (ascorbate), glutathione and total phenolics, and activities of the antioxidant enzymes superoxide dismutase (SOD, EC 1.15.1.1), catalase (CAT, EC 1.11.1.6), glutathione reductase (GR, EC 1.8.1.7), glutathione peroxidase (GPOX, EC 1.11.1.9) and ascorbate peroxidase (APOX, EC 1.11.1.11). The bioprotective capacity of the shoots of 7-day old PEF-treated or untreated seedlings were compared, by measuring the total radical scavenging capacity of shoot extracts, using a chemical (DPPH) assay, and by pre-culturing human intestinal Caco-2 cells with simulated gastrointestinal digests of the shoots of PEF-treated or untreated seeds, and then exposing the Caco-2 cells to H_2_O_2_-induced oxidative stress. The health of the Caco-2 cells was then assessed using standard methodologies.

## Results

### Effects of seed water content at the time of PEF treatment on wheatgrass seedling development

**Figure 1:**
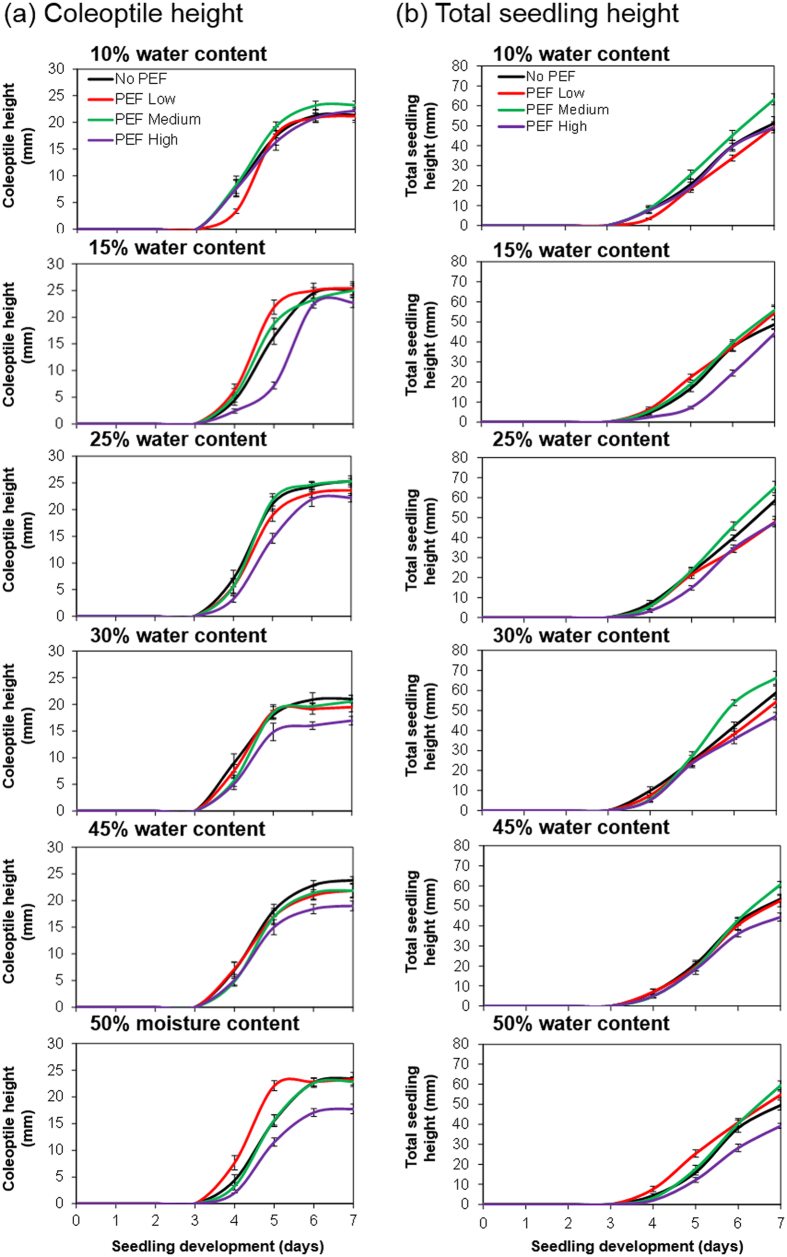
Growth performance over a 7 day period of (**a**) coleoptiles and (**b**) wheatgrass seedlings from seeds imbibed to different water contents at the time of PEF treatment at 0 kV/cm (No PEF), 0.5 kV/cm (PEF Low), 1.4 kV/cm (PEF Medium) or 2 kV/cm (PEF High). Error bars represent the standard error of the mean from fifteen independent wheatgrass seedlings (n = 15).

This study monitored the growth performance of wheatgrass seedlings from untreated seeds and from PEF-treated seeds with different water contents (Fig. 1). All seedlings showed similar developmental profiles, irrespective of treatment, with over 90% seed germination, good radical growth, and coleoptiles emergence 3 days after seed imbibition. The average height of 7-day old wheatgrass seedlings ranged between 50 and 65 mm. The 0.5 kV/cm PEF treatment of seeds did not influence seedling growth consistently, compared to untreated seedlings. However, it is interesting to note that seedlings produced from seeds treated at an electric field strength of 1.4 kV/cm were slightly larger than seedlings from untreated seeds. In contrast, PEF treatment of seeds at 2 kV/cm reduced coleoptile and primary leaf growth by at least 6 mm and 10 mm respectively, as compared to seedlings from untreated seeds.

### Changes in the levels of bioactive compounds and antioxidant enzyme activities in the shoots of wheatgrass seedlings from untreated or PEF-treated seeds. 

Wheatgrass seedlings were found to be rich in vitamin C and phenolics, with the total vitamin C and total phenolic contents of 7-day old wheatgrass shoots averaging 21.14 ± 1.82 μg/g fresh weight (FW) and 15.45 ± 0.16 mg/g FW, respectively. The total vitamin C and phenolic contents were not significantly influenced by any PEF treatment. However, it is interesting to note that while the total vitamin C content of shoots was not influenced by PEF treatment the proportion of the vitamin C pool in the reduced form (L-ascorbic acid) was up to 2-fold greater (10.51 vs 23.45 μg/g FW for untreated and PEF-treated seedlings respectively) in the shoots of seedlings produced from 2 kV/cm PEF-treated seeds, with water contents of 45% FW or greater, as compared to untreated seeds. There were no statistically significant differences in the levels of glutathione, and the activities of the antioxidant enzymes SOD, CAT, GPOX, GR and APOX in the shoots of wheatgrass seedlings from seeds exposed to PEF treatment at 0.5 kV/cm or 1.4 kV/cm compared to the shoots of seedlings from seeds not PEF-treated, at any of the water contents tested (data not shown). This indicates that PEF treatment of seeds and electric field strengths at or below 1.4 kV/cm had no prolonged influence on antioxidant metabolism in wheatgrass seedlings.

**Figure 2:**
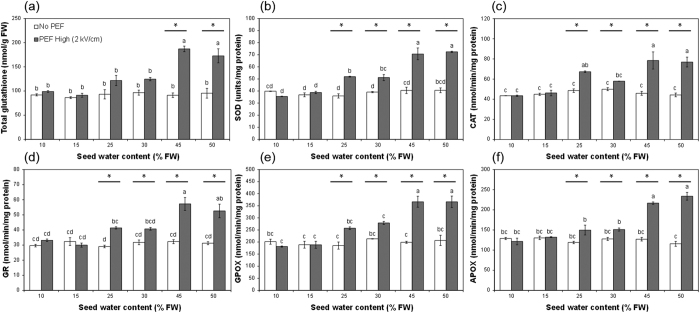
The influence of seed water content and PEF treatment at 2 kV/cm (PEF High) on the (**a**) total glutathione content and the activities of the enzymes (**b**) SOD, (**c**) CAT, (**d**) GPOX, (**e**) GR and (f) APOX in 7-day old wheatgrass seedlings. Error bars represent the standard error of the mean (n = 6). Different letters indicate statistically significant differences (*p* < 0.05) between the means. Significant differences (*p* < 0.05) between control and PEF treated seedlings are indicated with an asterisk (*).

However, a statistically significant (*p* < 0.05) increase in the total glutathione content was found in seedlings produced from 2 kV/cm PEF-treated seeds at water contents of 45 and 50% FW (Fig. 2a). In addition, a significant (*p* < 0.05) stimulation of the activities of all the antioxidant enzymes assayed was observed in seedlings from seeds treated at the high intensity electric field strength of 2 kV/cm (PEF High) with, for most enzymes, seed water contents of 25% FW or greater, compared to seedlings from seeds not PEF-treated (Fig. 2b–f). The increases in enzyme activities appeared to be dependent upon the water content of the seed when it was PEF-treated, with greater activities observed in seeds with higher water contents at the time of PEF treatment (Fig. 2b–f).

### The influence of PEF on the bioprotection capacity of wheatgrass shoots

Evaluation of the influence of PEF on the total antioxidant capacity of wheatgrass shoot extracts using the DPPH radicals scavenging assay, a chemical assays, showed equivalent scavenging capacities of between 70 and 80% towards DPPH radicals (data not shown) for of the shoot digests tested, irrespective of PEF treatment. Interestingly, no improvement in DPPH scavenging activity was observed in shoot digests from the seedlings of seeds PEF treated at 2 kV/cm and with a water content of 25% FW or greater, despite a significant (*p* < 0.05) increase in the total glutathione content and the activity of antioxidant enzymes. Only a weak correlation (r^2^ = 0.01–0.03; *p* < 0.05) between the results of the DPPH assay and the contents of endogenous antioxidants and antioxidant enzymes was found (Table 1).

**Figure 3:**
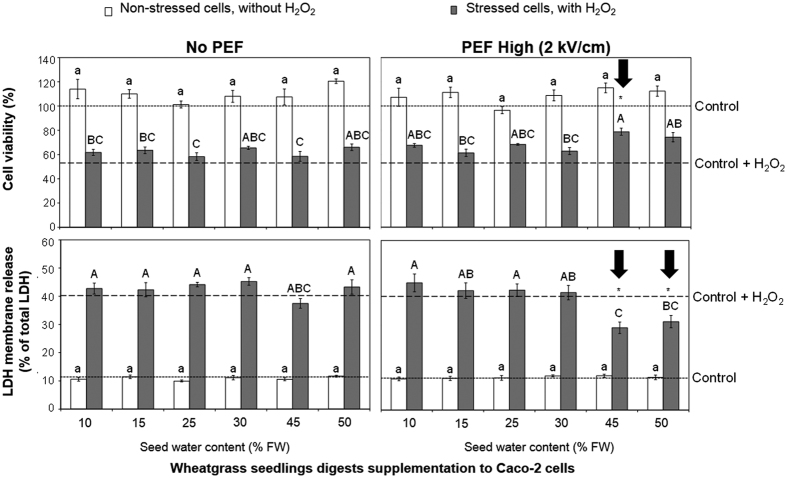
The ability of wheatgrass seedling digests, produced from untreated and “PEF High” treated seeds, in protecting Caco-2 cells from H_2_O_2_ exposure. Error bars represent the standard error of the mean from four independent cell culture experiments (n = 4) for each wheatgrass seedlings digest. Short and long dash lines represent the reference value for the untreated cells (control) and untreated cells with H_2_O_2_ exposure (control + H_2_O_2_) respectively. Different letters indicate statistically significant differences (*p* < 0.05) between the means. Significant differences (*p* < 0.05) comparing No PEF and PEF High seedling digests with differing water contents, are indicated with an asterisk (*).

The potential bioprotective capacity of wheatgrass seedling digests was evaluated by a biological assay using Caco-2 cells. The ability of wheatgrass digests to protect Caco-2 cells from H_2_O_2_-induced damage was determined using the MTT assay and the LDH leakage assay. The extent of protection afforded to the Caco-2 cells against H_2_O_2_ exposure was greater for wheatgrass digests produced from the shoots of seedlings whose seeds were treated with PEF at 2 kV/cm at seed water contents of 45 and 50% FW (Fig. 3). Pre-treatment of Caco-2 cells with these wheatgrass digests significantly (*p* < 0.05) increased cell viability (MTT assay) and reduced LDH leakage following exposure to H_2_O_2_. With respect to the two biomarkers of cellular health, digests of wheatgrass shoots from seeds treated with PEF at 0.5 and 1.4 kV/cm, regardless of the seed water content, did not provide greater protection to Caco-2 cells exposed to H_2_O_2_ than those from untreated seeds (data not presented). Providing the seed water content at the time of PEF treatment is 45% FW or greater, PEF treatment of seeds at 2 kV/cm (PEF High) has the potential to produce wheatgrass seedlings with a higher bioprotective capacity. Results from the MTT cell viability and LDH leakage assays of Caco-2 cells under H_2_O_2_ stress were found to be significantly correlated to glutathione levels (r^2^ = 0.30–0.33; *p* < 0.05) and antioxidant enzyme activities in the seedlings (r^2^ = 0.26–0.35; *p* < 0.05) (Table 1).

### The nutritional properties of shoots of wheatgrass seedlings from untreated or PEF-treated seeds

A nutritional analysis of the shoots of wheatgrass seedlings from untreated or PEF-treated seeds (45% FW; PEF High) showed that the nutritional profiles of both groups of shoots were not statistically different (Table 2). Hence PEF treatment of seeds did not reduce the nutritional value of the resultant seedlings.

## Discussion

This study explored the potential of PEF treatment of partially imbibed seeds to alter the metabolism of the resultant seedlings, using wheatgrass seeds as a model system. A negative impact of PEF at high intensities was reported for naked barley seeds^24^ and an electric field strength of 18 kV/cm was shown to delay the growth of yellow nutsedge seedlings^25^. However, Dymek *et al.*^14^ demonstrated that PEF treatment of barley seeds at field strengths below 1.2 kV/cm did not impair the metabolic activity of the embryo/seed during germination. Our results confirm that PEF treatment of seeds at field strengths of 1.4 kV/cm or less did not significantly influence the germination or growth potential of wheatgrass seeds or seedlings respectively, nor induce any sustained changes in antioxidant metabolism in seedlings from PEF-treated seeds. The influence of PEF treatments on seedling growth and gross metabolic activity has been investigated in other studies with variable results. When *Arabidopsis thaliana* seeds were subjected to nanosecond pulsed electric field an increase in average leaf area was observed in seedlings a few days after germination and was maintained for 2 weeks^12^. Growth promotion was observed in lettuce seedlings from seeds treated at PEF field strengths from 0.2 to 1.0 kV/cm, while those from seeds treated at PEF field strengths above 1.0 kV/cm showed growth inhibition, compared to controls^13^. In contrast, radicle growth was reduced by PEF treatment in germinating barley grains^14^. No gross changes in metabolic activity, other than a decrease in α-amylase concentration, were observed nor were significant changes in 2-DE protein profiles observed^14^.

The present study clearly demonstrated that, providing the water content of the seed at the time of PEF treatment was 25% FW or greater, PEF treatment of wheatgrass seeds at an electric field strength of 2 kV/cm stimulated an increase in antioxidant metabolism, with increased glutathione levels and increases in the activities of several enzymes associated with antioxidant metabolism in plant cells, with only a slight inhibition of growth potential. Maximal stimulation of antioxidant metabolism by PEF treatment was achieved in seedlings from seeds with water contents of 45 or 50% FW at the time of PEF treatment. Sabri *et al.*^18^ hypothesised that the cell membrane electropermeabilisation caused by PEF treatment could, to some extent, influence plant cell metabolism and that this could cause excessive production of ROS and thus induce oxidative stress.

While in the present study we did not directly measure ROS production or oxidative damage, our results clearly demonstrate that PEF treatment of appropriately hydrated seeds can induce a sustained up-regulation of antioxidant defences, a response indicative of oxidative stress in plant cells^26^. Further evidence of an apparent stress response was the reduced growth potential of seedlings from hydrated 2 kV/cm PEF treated seeds, over the 7-day duration of our study. An important finding of this study was the influence of seed water content, at the time of PEF treatment, on the response of the resultant seedlings. This is most probably due to the fact that seeds with higher water contents allow better distribution of the pulsed electric field around or into the seed, since the electrical resistance of the seed would be reduced as the seed water content increased^27^. Hence the embryo contained within the seed would be exposed to greater membrane permeabilisation/electrostimulation as a result of the PEF treatment.

From the results of the present and other studies it is clear that the application of PEF technology to appropriately hydrated seeds has the potential to modify the growth and stress responses of the resultant seedlings, and providing the correct parameters are chosen, PEF treatment of seeds can cause significant and sustained changes in seedling metabolism. Therefore PEF treatment of hydrated seeds potentially has a range of practical applications, including the production of sprouts with altered nutritional properties.

It is well known that wheatgrass shoots contain significant amounts of vitamin C (ascorbate), chlorophyll, phenolics and flavonoids, all with potent antioxidant properties^28^ and so we investigated whether extracts from the shoots of seedlings from PEF-treated seeds, with enhanced antioxidant metabolism, had a greater total antioxidant capacity than extracts from shoots of seedlings from seeds not treated with PEF (controls), with normal antioxidant metabolism. In addition, we investigated whether simulated gastrointestinal digests produced from PEF-enhanced shoots could provide greater protection against oxidative damage, using Caco-2 cells as a model system, than digests from control shoots.

No significant differences in the total antioxidant capacity, as determined using the DPPH scavenging capacity assay, were found when extracts from the PEF-enhanced shoots were compared to extracts from control shoots, despite the significant changes in shoot antioxidant metabolism observed. The most likely reason for this is the presence of relatively high levels of chlorophyll, vitamin C, phenolics and flavonoids in the extracts that would mask any increase in the size of the glutathione pool, which although the potential biological importance of glutathione would contribute relatively little to the total antioxidant capacity of the shoot extracts. Also, the higher level of antioxidant enzymes found in the PEF-enhanced shoots would not contribute to an increased total antioxidant capacity as determined by the DPPH assay. DPPH is a synthetic and a non-physiologically relevant radical^29^ and the DPPH assay simply measures the ability of an extract to scavenge free radicals via an electron transfer based chemical reaction mechanism^30^. The DPPH and other similar assays do not measure the contribution of enzymatic antioxidants towards ROS scavenging in living organisms, nor the influence that a complex mixture of molecules, as found in simulated gastrointestinal digests of plant materials, has on living biological systems exposed to the digests. Results from the present study clearly show that the DPPH assay is not a sensitive and reliable experimental approach to fully evaluate the potential of plant materials to protect cells from oxidative damage.

In contrast to the results from the DPPH assays, treatment of Caco-2 cells with simulated gastrointestinal digests from the PEF-enhanced shoots demonstrated a significantly increased capacity to cope with H_2_O_2_-induced oxidative stress, compared to Caco-2 cells treated with digests from control shoots. Treatment of Caco-2 cells with digests from PEF increased cell viability and decreased LDH activity following H_2_O_2_-induced oxidative stress, as assessed by the MTT assay indicating mitochondrial functionality^31^ and the LDH leakage assay for cell membrane integrity^32^, and hence demonstrated an enhanced bioprotective capacity compared to digests from control shoots. A correlation analysis demonstrated a significant positive correlation between the MTT assay and LDH leakage assay data and the total glutathione level and antioxidant enzyme activity data. Therefore, it can be concluded that the greater bioprotective capacity of the digests from the PEF-enhanced shoots was either directly or indirectly related to PEF-induced changes in seedling metabolism associated to oxidative stress tolerance.

While the focus of the present study was not to investigate the mechanisms by which digests from the PEF enhanced shoots protect Caco-2 cells from H_2_O_2_-induced oxidative damage the following provides some possible explanations for our results and some areas that warrant further investigation. In addition to antioxidants many other potentially bioactive plant metabolites are likely to be taken up and metabolised by the Caco-2 cells during the incubation step, prior to H_2_O_2_ exposure. In addition to acting directly as antioxidants these metabolites could act individually or synergistically, via complex signal transduction pathways, to enhance the Caco-2 cells own defences prior to H_2_O_2_ exposure. For example, Rodríguez-Ramiro *et al.*^33^ suggested that dietary flavanols might not only protect Caco-2 cells against an induced oxidative stress and subsequent cellular death by directly scavenging free radicals, but also by preventing caspase-3 activation and by activating cellular the Caco-2 cells own antioxidant defences, e.g. increasing the activities of antioxidant/detoxification enzymes. The fact that PEF treatment of seeds did not change the nutritional value of wheatgrass shoots, but increased the bioprotective potential of the shoots, clearly demonstrates that the influence of the PEF treatment used in the present study was positive and therefore PEF is of value for the development of functional foods.

It is also critical when developing technology-based techniques, such as PEF, to enhance the bioprotective capacities of plant-based food products that the methods used to assess any enhancements not only consider variations associated with the technology itself, e.g. field strengths, but also consider the physiological/developmental status of the living plant material. Very careful consideration should also be given to the methods used to assess the bioprotective capacity of the end product, as simple chemical or analytical assays may over- or under-estimate the actual bioprotective capacity of the end product in living systems. Induced defensive mechanisms cannot be measured or accurately predicted by indirectly, but can only be assessed using living cells.

## Conclusion

The present study is the first to investigate the use of PEF technology to manipulate a living plant system, in this study seeds, with the aim of enhancing the functional characteristics of a plant-based food. The seed water content at the time of PEF treatment was found to be a very important factor to consider, as variations in seed water content influenced the ability of a PEF treatment to induce significant and sustained changes in the metabolism of the resultant wheatgrass seedling. When seeds were appropriately hydrated, subsequent electrostimulation by PEF increased the glutathione content and the activities of antioxidant enzymes in the resultant seedlings, enhancing the bioprotective capacity of harvested shoots. “Electropriming” of seeds using PEF, if appropriately optimised, could be used as a relatively simple method to produce high-quality and nutritious sprouts without using chemical treatments. While the present study concentrated on the potential of PEF treatment of imbibed seeds for the production of wheatgrass shoots with greater bioprotective capacity for human consumption, electrostimulated shoots could also be of value as a supplement for animal feeds.

## Methods

**Figure 4:**
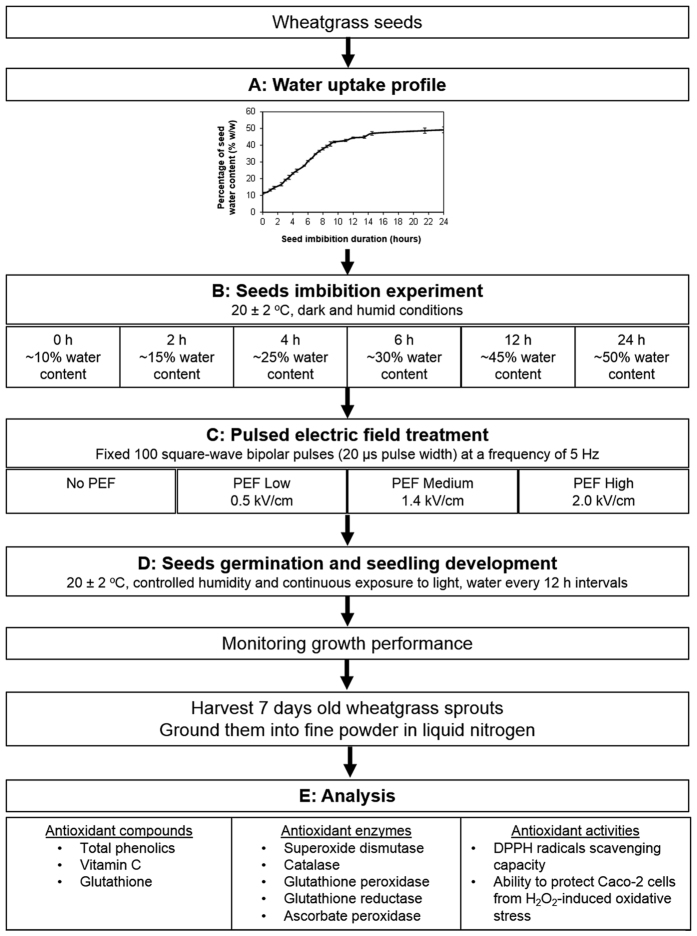
Schematic overview of the experimental design.

Figure 4 illustrates the schematic overview of the experimental design used in this study, of which further details regarding seeds preparation and imbibition, PEF treatment conditions, development conditions of wheatgrass seedlings and the relevant analysis are described below.

### Preparation of seeds

Seeds of wheatgrass (*Triticum aestivum* L.) purchased from Kings Seeds (Katikati, Bay of Plenty, New Zealand) and were selected from a single batch for uniformity of shape, size and colour. Damaged seeds were not used in this study.

### Determination of the seed water uptake

Seed water uptake experiments were performed by weighing lots of 20 seeds and placing each seed lot (n = 3) into a 9-cm petri dish containing a single layer of filter paper (Munktell and Filtrak GmbH, Niederschlag, Germany; Grade 292) and enough water to half-fill the dish. The seeds were allowed to imbibe and germinate at 20 ± 2 ^o^C in the dark condition (Jeio Tech IB-15G, Acorn Scientific Ltd, Auckland, New Zealand), and the percentage germination (emergence of a 1 mm long radicle) was determined. Water uptake by the seeds was determined gravimetrically as a function of imbibition time. For the determination of water uptake, seeds were removed at predetermined times, briefly wiped free of the surface water with paper tissue, weighed using an electronic balance (Kern & Sohn GmbH, Balingen, Germany; error of ±0.1 g), returned to the petri dish and reweighed at the next desired time point.

Seed dry weights were determined by drying seed lots in a convection oven (Qualtex Solidstat Universal series 2000, Watson Victoria Ltd, Dunedin, New Zealand) at 60 °C for at least 48 h or until a constant weight of dry matter was achieved. Results correspond to the means of 3 seed lots and seed water contents are expressed as percentage water content on a fresh weight basis (% FW). The initial water content of dry seeds prior to imbibition was approximately 10% FW. Seeds, imbibed for 2, 4, 6, 12 and 24 h had water contents of 15, 25, 30, 45 and 50% FW respectively (Fig. 4), were used for subsequent PEF experiments.

### Pulsed electric field (PEF) treatments

Wheatgrass seeds not imbibed (10% FW water content) or imbibed to water contents of 15, 25, 30, 45 and 50% FW, were divided into batches to be used for the PEF experiments. Control batches without PEF treatment are referred as “No PEF”. For the PEF treatments the ELCRACK-HVP 5 (German Institute of Food Technologies, Quakenbrück, Germany) of non-continuous configuration was used. The batch treatment chamber (100 mm length × 80 mm width ×50 mm height, 400 mL capacity) consisted of two parallel stainless steel electrodes of 5 mm thickness separated by a distance of 80 mm. For each PEF treatment, the treatment chamber was filled with 20 g seeds (in FW) and 80 g distilled water was added to fully submerge the seeds and enable uniform distribution of electric field strength during PEF treatment. In this study, different input operating variables were used that resulted in effective electric field strengths of 0.5 (referred as “PEF Low”), 1.4 (referred as “PEF Medium”) and 2 kV/cm (referred as “PEF High”) for 100 pulses with a pulse width of 20 μs at a fixed pulse frequency of 5 Hz. The specific energy inputs, calculated according to Zhang *et al.*^34^, were 0.26 ± 0.01 kJ/kg, 0.71 ± 0.01 kJ/kg and 1.54 ± 0.03 kJ/kg for “PEF Low”, “PEF Medium” and “PEF High” respectively. A nearly square wave bipolar pulse was observed on-line with oscilloscope (Model UT2025C, Uni-Trend Group Limited, China) for every PEF treatments. All treatments were conducted at an ambient temperature (20 ± 2 ^o^C).

### Seeds germination and seedlings growth

Seeds from the various treatments were sown evenly on a single layer of chromatography paper (Whatman, Grade 3030917), saturated with deionised water, in individual plastic boxes (155 × 155 × 86 mm) and a further 30 ml of deionised water was added and the seeds were incubated at 20 ^o^C in the dark. Irrespective of the duration of imbibition prior to PEF treatment, seeds were imbibed for a total of 24 h at 20 ^o^C in the dark. After this time the seeds were transferred to Contherm 620 (Contherm Scientific Ltd, Wellington, New Zealand) plant-growth chambers and the resultant seedlings were allowed to grow and develop for a further 6 days, at 20 ± 2 ^o^C, a relative humidity of 70%, with continuous exposure to light (150 μmol m^2^ s^−1^ PAR). The seedlings were routinely watered every 12 h with appropriate amount of deionised water. For each treatment the shoot length of fifteen (n = 15) seedlings were measured with ruler to the nearest mm at 24 h intervals. For each treatment the green shoots of 200 seedlings, of similar sizes, were harvested 7 days after seeds were sown, by the shoot approximately 10 mm above the seed remains and randomly divided into two duplicate lots of 100 seedlings. Each lot of 100 shoots was then frozen in liquid nitrogen, ground to fine powder using a mortar and pestle chilled in liquid nitrogen and the ground frozen tissue was immediately stored at −80 ^o^C for further analyses.

### Determination of the total phenolics content

Total phenolics were assayed using Folin-Ciocalteu method of Ainsworth and Gillespie^35^, with minor modifications. The sample extract was prepared by homogenising frozen powdered shoot tissue (0.1 g) with 900 μL of methanol, followed by centrifugation for 2 min at 21000 *g* (IEC Micromax, Massachusetts, USA). Extraction was performed in triplicates for each sample. One millilitre of Folin-Ciocalteu reagent (10% v/v) was added to the sample extract and mixed gently. The mixture was allowed to stand at room temperature (25 ± 2 ^o^C) for 5 min before addition of 1 mL of sodium carbonate (7.5% w/v). After standing at room temperature for 60 min, the absorbance of the mixture was read at 765 nm using a spectrophotometer (Ultrospec 3300 Pro, Amersham Biosciences, Uppsala, Sweden). The standard calibration curve was plotted using gallic acid. The results were expressed as milligrams of gallic acid equivalents (GAE) per gram of wheatgrass seedlings on a fresh weight (FW) basis.

### Determination of the vitamin C content

Vitamin C extraction was carried out as described by Leong and Oey^36^. Frozen powdered shoot tissue (0.2 g) was homogenised with 1 mL of cold extraction buffer (5% (w/v) metaphosphoric acid, pH 4, containing 1 mM EDTA). The homogenate was centrifuged for 2 min at 21000 *g* (IEC Micromax). The supernatant was collected and then filtered. Extraction was performed in triplicates for each sample. The sample extracts were injected into a reversed-phase Prevail C_18_ column (5 μm, 250 × 4.6 mm; Grace Davison Discovery Sciences, Deerfield, Illinois, USA). Vitamin C content was estimated after pre-column reduction using reducing agent of tris-[2-carboxyethyl]-phosphine hydrochloride (2.5 mM; dissolved in 5% metaphosphoric acid (pH 6) containing 1 mM EDTA) for at least 10 h incubation at 4 ^o^C. An injection volume of 50 μL was used to quantify vitamin C content. The elution was undertaken isocratically (Agilent 1200 system; Massachusetts, USA) using a mixture of 90% formic acid (0.1% v/v) and 10% methanol at a flow rate of 0.8 mL/min for a total elution time of 30 min. Vitamin C was identified based on peak purity using a diode array detector and retention time between 4.3 and 4.4 min. The quantification was performed at 245 nm and 25 °C. Vitamin C content was estimated based on the external L-ascorbic acid (L-AA) standard solution (dissolved in 5% metaphosphoric acid (pH 4) containing 1 mM EDTA). The results were expressed as micrograms of L-AA equivalents per gram of wheatgrass seedlings on a fresh weight (FW) basis.

### Determination of the glutathione content

Glutathione was extracted by homogenising frozen powdered shoot tissue (0.1 g) in 900 μL of cold extraction buffer (0.6% (w/v) sulfosalicylic acid-Triton X-100). The extract was centrifuged (Eppendorf 5417R, Eppendorf South Pacific Pty. Ltd., North Ryde, NSW, Australia) at 20800 *g* and 4 ^o^C for 30 min. Extraction was performed in triplicates for each sample. The supernatant was then collected and stored frozen at −80 ^o^C until glutathione analysis. Glutathione and glutathione disulphide levels were measured using the enzymatic recycling method in a microplate assay as described by Rahman *et al.*^37^. Assays were carried out using a Perkin Elmer (Wallac Victor) 1420 multilabel counter (PerkinElmer, San Jose, California, USA) controlled by a PC, and fitted with a temperature control cell, set at 25 ^o^C, and an auto-dispenser. Data were acquired and processed using the WorkOut 2.0 software package (PerkinElmer, San Jose, California, USA). The results were expressed as nanomoles of glutathione per gram of wheatgrass seedlings on a fresh weight (FW) basis.

### Determination of the antioxidant enzyme activity

#### Activity of superoxide dismutase (SOD), catalase (CAT), glutathione peroxidase (GPOX) and glutathione reductase (GR)

SOD, CAT, GPOX and GR were extracted on ice as followed: 950 μL of ice-cold extraction buffer (100 mM potassium phosphate (pH 7.5), 0.25 mM K_2_EDTA, 1 mM PMSF and 2% (w/v) Polyclar AT) (SERVA Chemicals Limited, Heidelberg, Germany)) was added to frozen powdered shoot tissue (50 mg) and mixed with a vortex mixer. The homogenate was then centrifuged (Eppendorf 5417R) at 20800 *g* and 4 ^o^C for 30 min. Extractions were done in triplicate for each sample. The supernatant was then collected, divide into aliquots and stored at −80 °C before performing the enzyme assays as detailed below. The total protein content of each enzyme extract was determined using the modified Lowry protein assay, result expressed in milligram of total protein^38^. Extracts were diluted as required for the enzyme assays detailed below and all enzyme assays were carried out using a Perkin Elmer (Wallac Victor) 1420 multilabel counter controlled by a PC, and fitted with a temperature control cell, set at 25 ^o^C, and an auto-dispenser. Data were acquired and processed using the WorkOut 2.0 software package (PerkinElmer).

The SOD assay was conducted based on the work of Banowetz *et al.*^39^ with minor modifications. Sample extract (50 μL) or SOD standards (bovine liver SOD, Sigma Aldrich, St. Louis, MO, USA) was mixed with 125 μL of freshly prepared reaction buffer (pH 7.8) containing piperazine-1,4-bis(2-ethanesulphonic acid, 0.4 mM *o*-dianisidine, 0.5 mM diethylenetriaminepentaacetic acid, and 26 μM riboflavin. The absorbance at 450 nm (A_450_) was measured immediately (t = 0 min) and samples were illuminated with an 18 W fluorescent lamp placed 12 cm above the plate for 30 min (t = 30 min) and the absorbance was measured again at 450 nm. A linear regression curve was used to prepare a standard line relating SOD activity to the change in A_450_. The SOD activities in the sample extracts, calculated with reference to the standard line, were expressed as units SOD per milligram of total protein. One unit of SOD activity corresponded to the amount of enzyme that inhibited the reduction of cytochrome *c* by 50% in a coupled system with xanthine oxidase at pH 7.8 and 25 °C.

CAT was assayed using the method of Summermatter *et al.*^40^, as adapted by Gillespie *et al.*^41^ for use in a microplate reader. Briefly, 50 μL of enzyme extract was mixed with 50 μL of sodium phosphate buffer (50 mM, pH 7), after which 50 μL of hydrogen peroxide (H_2_O_2_, 35 mM) was added to each well. Duplicate samples of each extract were incubated at 25 ^o^C and after 1 min; the reactions were stopped in one of samples by the addition of 50 μL 15% (w/v) trichloroacetic acid (TCA). After a further 2 min, the reactions were stopped in the second set of samples by the addition of TCA as described above. The H_2_O_2_ remaining after incubation with CAT was determined by mixing 5 μL of the reaction mix with 100 μL of H_2_O_2_ determination reagent (contain 1 g/L 2,2′-azino-bis(3-ethylbenothiazoline-6-sulphonic acid, 0.8 U/mL horseradish peroxidase from Sigma) in the well of a new microplate, incubating the plate for 10 min at 20 ^o^C, and measuring the absorbance at 410 nm. The amount of H_2_O_2_ was determined using standard curves obtained with known concentrations of H_2_O_2_. Purified bovine liver CAT (Sigma) in homogenisation buffer was used as a positive control. CAT activities in the extracts were expressed as nanomoles of H_2_O_2_ consumed per min per milligram of total protein.

GPOX activity was measured according to the spectrophotometric method^42^ with modifications for use in a plate reader. Briefly, 20 μL of extract or standard (bovine erythrocytes GPOX from Sigma) was mixed with 170 μL of reaction buffer containing 50 mM Tris-HCl buffer (pH 7.6), 5 mM EDTA, 0.14 mM nicotinamide adenine dinucleotide phosphate (NADPH), 1 mM glutathione and 3 U/mL wheat germ GR (Sigma). The reaction was initiated by the addition of 20 μL *t*-butyl hydroperoxide to give a final concentration of 0.2 mM. The consumption of NADPH was monitored at 340 nm every 30 sec intervals for 3 min, with the plate shaken automatically before each reading was taken. GPOX activities in the extracts were calculated with reference to a standard line constructed with GPOX purified from bovine erythrocytes. GPOX activities in the extracts are expressed as nanomoles of glutathione consumed per min per milligram of total protein.

GR was assayed with minor modifications from previous work described^43^. The assay were carried out by mixing 50 μL enzyme extract or standards (GR from germ wheat, Sigma) with 150 μL of sodium phosphate buffer (100 mM, pH 7.6) containing 0.1 mM 5,5′-dithiobis(2-nitrobenzoic acid) and 10 μL of NADPH (10 mg/mL, 12 mM). The reaction was imitated by the injection of 10 μL of oxidised glutathione (1 mg/mL, 3.25 mM) and the absorbance at 415 nm was measured every 30 sec intervals for 3 min, with the plates shaken automatically before each reading was taken and recorded. The rate of increase in absorbance at 415 nm per min was calculated and a regression analysis was used to prepare a standard line relating standard GR activities to the change in the absorbance at 415 nm. GR activities in the extracts, calculated with reference to the standard line, were expressed as nanomoles of oxidised glutathione reduced per minute per milligram of total protein.

#### Ascorbic acid peroxidase (APOX) activity

APOX extraction was performed by adding 950 μL ice-cold extraction buffer (100 mM potassium phosphate (pH 7.5), 0.25 mM K_2_EDTA, 5 mM ascorbate and 2% (w/v) Polyclar AT to frozen wheatgrass seedlings powder (50 mg) and mixed with a vortex mixer. The homogenate was then centrifuged (Eppendorf 5417R) at 20800 *g* and 4 ^o^C for 30 min. Extraction was done in triplicates for each sample. The supernatant was then collected, divide into aliquots and stored at -80 °C before performing the APOX assay as detailed below. The total protein content of each enzyme extract was determined using the modified Lowry protein assay, result expressed in milligram of total protein^38^.

The APOX assay was conducted according to the method of Nakano and Asada^44^, by following the decrease in the absorbance at 290 nm as ascorbate disappeared, with modifications for use in a microplate reader^45^. Briefly, 50 μL enzyme extract was mixed with 200 μL of potassium phosphate (100 mM, pH 7), 0.5 mM L-AA, 0.2 mM H_2_O_2_ and 1 M EDTA in a UV transparent microplate (Greiner Bio-one GmbH, Kremsmünster, Austria). The absorbance at 290 nm was measured at every 30 sec intervals for 3 min. corrections were made for the oxidation of L-AA in the absence of H_2_O_2_ and for the low, non-enzymatic oxidation of L-AA by H_2_O_2_ when necessary. APOX activities were calculated using an extinction coefficient of 2.8 mM^−1^ cm^−1^, corrected for the calculated path length of the solution in the microplate (0.6 cm). APOX activities in the extracts were expressed as nanomoles of ascorbate reduced per minute per milligram of total protein.

### Determination of the DPPH radical scavenging capacity

The sample extract prepared for quantification of total phenolics was used to measure the total antioxidant capacity. The sample extract was added in a 96-well plate (Greiner Bio-One GmbH) followed by the addition of 2, 2-diphenyl-1-picrylhydrazyl (DPPH) solution (0.2 mM in methanol). The assay was run using a microplate reader (Synergy 2; BioTek Instruments, Vermont, USA) fitted with a temperature control of 25 ^o^C and the plate was shaken for 10 sec before reading the absorbance. The decrease in absorbance at 516 nm was followed for 60 min and recorded with Gen5 data analysis software (BioTek Instruments, Vermont, USA). The control is the absorbance measured at 516 nm against methanol with DPPH. The antioxidant capacity was expressed as the percentage of DPPH inhibition when the decrease in absorbance reached a plateau (usually achieved within 30 to 45 min of 25 ^o^C incubation).

### Simulated *in vitro* human gastrointestinal digestion

*In vitro* digestion to simulate human gastric and intestinal digest in human body was carried out as outlined by Glahn *et al.*^46^ with minor modifications. Sodium phosphate buffer (10 mM, pH 7) containing α-amylase was added to the frozen wheatgrass seedlings powder and then incubated for 2 min at 37 ^o^C in a ProBlot hybridisation oven (Labnet International, New Jersey, USA). Afterwards, hydrochloric acid (0.1 M) solution was added to achieve pH 2 followed by addition of 1 mL of 40 mg/mL pepsin in hydrochloric acid (0.1 M) solution. The mixture was incubated for 1 h at 37 ^o^C in the hybridisation oven, shaking at a rate of 55 strokes per min to mimic digestion in human stomach. Then, sodium bicarbonate (1 M) solution was added drop wise to raise the pH of the digest to between 5 and 5.5. Then, 5 mL of simulated intestinal digestion fluid consisting of sodium bicarbonate (1 M), pancreatin (2 mg/mL) and bile salts (12 mM) were added into the mixture. The mixture was further incubated for 2 h at 37 ^o^C in a hybridisation oven, shaken at a rate of 55 strokes per min to simulate the human small intestine during digestion. As a final step, sodium hydroxide (1 M) solution was added to the digest to raise the pH to 7.4. The volume of the mixture was brought to 40 mL with sodium chloride (120 mM) solution. The mixture was then filtered through Whatman grade 1 filter paper (LabServ, Auckland, New Zealand) and stored in 1.5 mL aliquots at −80 ^o^C. These aliquots, referred as the “digests”, were ultrafiltered using Vivaspin 500 (10000 MWCO) ultrafiltration units (Sartorius Stedim Biotech GmbH, Göttingen Germany), according to the manufacturer’s instructions, to remove digestive enzymes prior to incubation with the Caco-2 cells.

### Cell culture experiments using Caco-2 cell lines

Human Caco-2 cell lines (HTB-37; American Type Culture Collection, Rockville, Maryland, USA) were cultured in Dulbecco’s modified Eagle’s medium (DMEM; Gibco, Grand Island, New York, USA), supplemented with 1% non-essential amino acids, 1% L-glutamine, 20% heat-inactivated fetal bovine serum (FBS), 100 units/mL of penicillin and 100 μg/mL of streptomycin in 50 cm^2^ plastic flasks (JET Bio-Filtration Products; Guangzhou, China). Cells were grown in a humidified atmosphere of 5% CO_2_ at 37 °C and the culture medium was replaced every 2 to 3 days. Cells were sub-cultured after reaching approximately 80% confluence, and used between passages 18 and 24 in all experiments.

#### Induction of oxidative stress by exposing to H_2_O_2_

Caco-2 cells were grown to confluence in 96 well microplates under the conditions detailed above. The culture medium was removed and replaced with fresh medium containing 25% (v/v) of the digests diluted with culture medium. Cells were cultured for 24 h, the culture medium was then removed and the Caco-2 cells were washed with fresh culture medium without FBS. The induction of oxidative stress was carried out by exposing Caco-2 cells to 500 μM hydrogen peroxide (H_2_O_2_) for 1 h. The untreated cells were taken as “control” whereas the untreated cells after addition of H_2_O_2_ were referred as “control + H_2_O_2_”. Four independent trials were conducted to determine the cell viability and lactate dehydrogenase membrane release after digest supplementation.

### Determination of the biomarkers for general cellular health and integrity

#### MTT cell viability assay

The 3-(4,5-dimethylthiazol-2-yl)-2,5-diphenyltetrazolium bromide (MTT) assay was used to measure cell viability. After treatment of the Caco-2 cells, the culture medium was removed and the cells were washed with fresh culture medium without FBS. Cells were then incubated with 0.5 mg/ml MTT, in culture medium without FBS, for 4 h at 37 °C in a 5% CO_2_ atmosphere. The medium was removed and the formazan crystals were dissolved in 10% sodium dodecyl sulphate in 0.01 M hydrochloric acid. The absorbance was then measured at 570 nm using a Perkin Elmer (Wallac) 1420 multilabel counter. Cell viability is expressed as a percentage of the control (untreated cells) value.

#### Lactate dehydrogenase (LDH) membrane release assay

The plasma membrane integrity of Caco-2 was determined by measuring the release of lactate dehydrogenase (LDH) from cells into the surrounding culture medium. After treatment 100 μL of culture medium was mixed with 100 μL of reaction cocktail (Cytotoxicity Detection Kit (LDH); Roche Diagnostics, Mannheim, Germany). The mixture was incubated for 30 min at 25°C and the absorbance measured at 490 nm using a PerkinElmer (Wallac) 1420 multilabel counter. LDH activity is expressed as a percentage of total LDH, from fully lysed cells (exposed to 2% Tween 80).

### Nutritional analysis

Shoot tissues were oven dried for 48 hours at 55 ^o^C and then ground to a fine power, prior to analysis by R. J. Hill Laboratories Limited (Hamilton, New Zealand), using near-infrared spectroscopy.

### Data analysis

The results are presented as means ± the standard error of the mean for at least 3 or 4 independent measurements. The student’s *t*-test was used for single comparisons or an analysis of variance (ANOVA) was used for multiple comparisons, followed by a post-hoc Tukey’s Honestly Significant Difference (HSD) test to compare the means with respect to PEF processing intensities and water contents. Correlations among the data obtained (n = 192) were calculated using Pearson’s correlation coefficient (*r*). The statistical significance of the correlation was evaluated by calculating two-tailed probability values (*p*-values). The criterion employed for a statistical significant difference was *p* < 0.05. All statistical analyses were performed using SPSS Statistics version 20 (IBM Corporation, New York, USA).

## Additional Information

**How to cite this article**: Leong, S. Y. *et al.* Electropriming of wheatgrass seeds using pulsed electric fields enhances antioxidant metabolism and the bioprotective capacity of wheatgrass shoots. *Sci. Rep.* **6**, 25306; doi: 10.1038/srep25306 (2016).

## Figures and Tables

**Table 1:** Summary of the coefficients of determination (r^2^) describing the linear relationships between the levels of antioxidants and antioxidant enzymes with markers of bioprotective capacity.

Parameters	Antioxidant activity markers		
	Chemical marker^1^	Biological markers^2^	
	DPPH scavenging capacity	MTT cell viability	LDH membrane release
Antioxidants			
Glutathione	0.0194	0.3324	0.3027
Vitamin C	0.0006	0.0056	0.0178
Total phenolics	0.0191	0.0209	0.0457
Antioxidant enzymes			
SOD	0.0271	0.3032	0.3201
CAT	0.0031	0.2642	0.2624
GPOX	0.0183	0.2933	0.3118
GR	0.0123	0.3107	0.3010
APOX	0.0036	0.3060	0.3465
DPPH^3^	–	0.0062	0.0114

Data presented as the square of Pearson’s correlation coefficient (r^2^) for which the correlations among data (n = 192) are statistical significance at *p* < 0.05. All experimental data (n = 192) were collated and analysed using linear regression (SPSS Statistics software).

^1^Based on the DPPH antioxidant activity assay.

^2^Based on biomarkers indicating the cellular health and integrity of Caco-2 cells.

^3^Linear correlation representing the variation in biomarkers to indicate cellular health and integrity can be explained by DPPH scavenging capacity.

**Table 2:** The nutritional profiles of shoots from untreated or PEF-treated seeds (45% FW; PEF High).

	Control	PEF
	(Mean +/− S.E.)	(Mean +/− S.E.)
Nitrogen (%DM)	3.30 +/− 0.27	3.17 +/− 0.20
Crude Protein (%DM)	20.43 +/− 1.72	19.80 +/− 1.40
Acid Detergent Fibre (%DM)	27.97 +/− 0.49	27.63 +/− 0.93
Neutral Detergent Fibre (%DM)	48.87 +/− 1.49	48.53 +/− 2.58
Ash (%DM)	8.93 +/− 0.78	9.13 +/− 0.89
Organic Matter (%DM)	91.07 +/− 0.79	90.87 +/− 0.90
Soluble Sugars (%DM)	13.37 +/− 0.24	13.47 +/− 0.94
Starch (%DM)	0.90 +/− 0.23	1.00 +/− 0.29
Crude Fat (%DM)	4.03 +/− 0.12	3.93 +/− 0.09
Metabolisable Energy (MJ/kgDM)	10.91 +/− 2.08	10.90 +/− 0.21
Non Structural Carbohydrate (%DM)	17.77 +/− 2.38	18.60 +/− 2.10

There were no statistically significant differences (n = 3; *p* < 0.01) between control and PEF treatments.
